# The complete chloroplast genome of *Tripterygium wilfordii* Hook. f. (Celastraceae)

**DOI:** 10.1080/23802359.2022.2119103

**Published:** 2022-09-23

**Authors:** Yuan Zhong, Jingzheng Zhang, Zhenzhen Bao

**Affiliations:** School of pharmacy, Jiangsu Health Vocational College, Nanjing, Jiangsu, P. R. China

**Keywords:** *Tripterygium wilfordii*, complete chloroplast genome, phylogenetic analysis

## Abstract

*Tripterygium wilfordii* is a perennial vine plant with medicinal value and belongs to the family of Celastraceae. In this study, we sequenced and analyzed the complete chloroplast genome of *T. wilfordii*. The chloroplast genome was 156,700 bp in length with a GC content of 37.47%. It contained two inverted repeat (IR) regions of 26,461 bp; each region was separated by large single-copy and small single-copy regions of 85,409 bp and 18,369 bp, respectively. In total, we annotated 134 unique genes, consisting of 89 protein-encoding genes, 8 rRNAs and 37 tRNAs. Phylogenetic analysis revealed that *T. wilfordii* was sister to *T. regelii* in a clade of *Tripterygiumii* species that was sister to a clade of *Euonymus* species.

*Tripterygium wilfordii* Hook.fil. (1862), a typical representative of the genus *Tripterygium*, is a perennial vine plant that is widely distributed in East Asia (Liu et al. 2010). The roots of *T. wilfordii*, named ‘*Leigongteng’* in Chinese, is used in traditional Chinese medicine. Pharmacological research has confirmed it has significant anti-inflammatory, immunosuppressive, antitumor, and other pharmacological benefits (Kang et al. 2021). Currently, several Chinese patent drugs derived from *T. wilfordii* have been approved by the China Food and Drug Administration for clinical usage in immunosuppression following organ transplantation and to treat autoimmune and inflammatory related diseases (Liu et al. 2020). However, wild *T. wilfordii* populations have diminished due to unregulated harvesting; therefore, it is necessary to develop genomic resources for this species to facilitate research. In this study, we sequenced and analyzed the complete chloroplast genome of *T. wilfordii*. This will provide useful information on the phylogeny and evolution of genus *Tripterygium* and aid studies to conserve the invaluable natural *T. wilfordii* populations.

The total genomic DNA was extracted by a modified CTAB method (Doyle and Doyle 1987) from fresh leaves of *T. wilfordii* collected from the Botanical Garden of Jiangsu Health Vocational College (Nanjing, China; N 32°5′2.333″, E 118°37′6.820″). A voucher specimen was deposited at the herbarium of Jiangsu Health Vocational College under the voucher number, FF20210720ZY-12 (https://www.jssmu.edu.cn/, contact person: Mr. Hu Xu, and email: 1827891673@qq.com). The entire genome sequencing was implemented by Bio&Data Biotechnologies Inc. (Guangzhou, China). Following DNA extraction, we fragmented 1 μg of purified DNA by ultrasound on Covaris E220 (Covaris, Brighton, UK) and used it to set up ∼300 bp short-insert libraries. These qualified libraries were sequenced with PE150 bp on an BGISEQ-500 sequencer (Hefei Bio&Data Biotechnologies Inc.) according to the manufacturer’s instructions detailed in the previous literature (Huang et al. 2017). In total, 35.82 Mb clean reads were obtained and assembled *de novo* using NOVOplasty 2.7.2 (Dierckxsens et al. 2017). Annotation was performed using CPGAVAS2 (Shi et al. 2019) and Basic Local Alignment Search Tool (BLAST) (Altschul et al. 1997) searches.

The chloroplast genome of *T. wilfordii* was 156,700 bp in length, containing two inverted repeat (IR) regions of 26,461 bp; each region was separated by large single-copy (LSC) and small single-copy (SSC) regions of 85,409 and 18,369 bp, respectively. A total of 134 functional genes were predicted, including 89 protein-coding genes, 37 transfer ribonucleic acid (tRNA) genes, and 8 ribosomal RNA (rRNA) genes. While most genes were in the single copy regions, 18 genes including 7 protein-coding genes, 4 rRNA genes, and 6 tRNA genes were duplicated in the IR regions. 19 genes had two exons and four genes (*clpP*, *ycf3*, and two *rps12*) contained three exons. The total sequenced GC content was 37.47%, while the corresponding values in the LSC, SSC, and IR regions were 35.34, 31.98, and 42.82%, respectively.

Alignment was carried out on the 27 chloroplast genome sequences using MAFFT version 7.0 to investigate phylogenetic relationships of *T. wilfordii* (Katoh and Standley 2013). A maximum likelihood (ML) tree was constructed using FastTree version 2.1.10 (Price et al. 2010). The results showed that among the Celastraceae species sampled, *T. wilfordii* was sister to *T. regelii* a clade containing all other sampled *Tripterygium* species. This clade was in turn sister to a clade containing the *Euonymus* species (Figure 1). The newly disclosed chloroplast genome MN624264 of *T. wilforgii* was annotated using the same method as in this study. While the OK065822 and MN624264 plastomes have the same number of genes, the latter has a total length of 158,916 bp and 37.5% GC content owing to a 938 bp indel that was not detected in the *T. wilfordii* plastome we describe here. In conclusion, the complete chloroplast genome sequence of *T. wilfordii* will be useful for future research in conservation genetics and molecular-assisted breeding.

**Figure 1.**
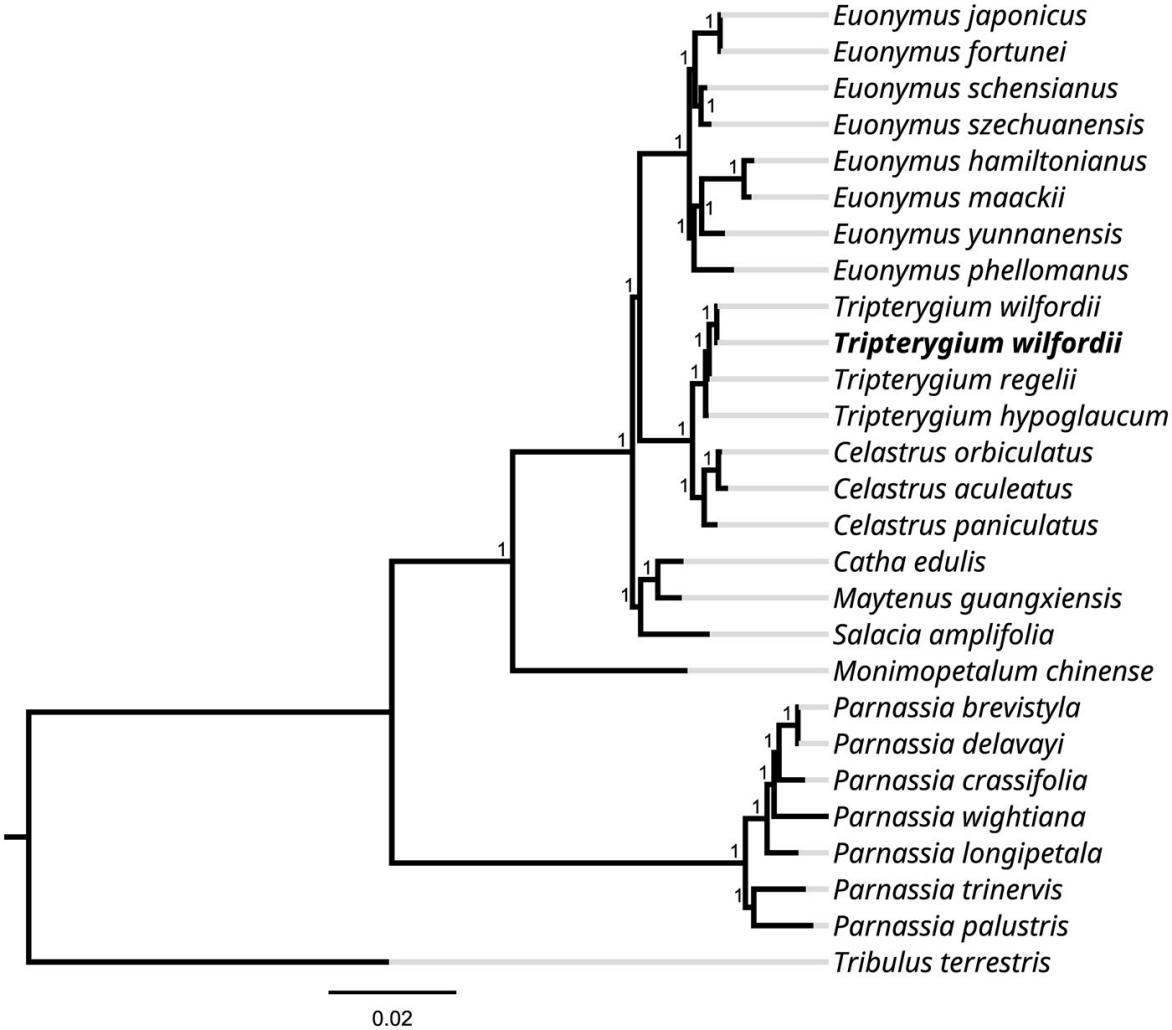
Phylogenetic tree inferred using the Maximum Likelihood (ML) method based on 27 representative species (with 1000 bootstrap repetitions). The following sequences were used: *Tripterygium wilfordii* OK065822 (in this study), *Euonymus japonicus* NC_028067 (Choi and Park 2016), *Euonymus fortunei* NC_057058 (unpublished), *Euonymus schensianus* NC_036019 (Wang et al. 2017), *Euonymus szechuanensis* NC_047463 (Wang et al. 2020), *Euonymus hamiltonianus* NC_037518 (unpublished), *Euonymus maackii* NC_057059 (unpublished), *Euonymus yunnanensis* MW770452 (unpublished), *Euonymus phellomanus* NC_057060 (unpublished), *Tripterygium wilfordii* MN624264 (unpublished), *Tripterygium regelii* MN624266 (unpublished), *Tripterygium hypoglaucum* MN624265 (unpublished), *Celastrus orbiculatus* MW316708 (unpublished), *Celastrus aculeatus* MW801026 (unpublished), *Celastrus paniculatus* OL804289 (unpublished), *Catha edulis* KT861471 (unpublished), *Maytenus guangxiensis* NC_047301 (Shi and Liu 2020), *Salacia amplifolia* NC_047214 (Lin et al. 2019), *Monimopetalum chinense* MK450440 (Pan et al. 2019), *Parnassia brevistyla* MG792145 (Xia et al. 2018), *Parnassia delavayi* MK580540 (unpublished), *Parnassia crassifolia* MK580538 (unpublished), *Parnassia wightiana* MN398191 (Li et al. 2019), *Parnassia longipetala* MK580539 (unpublished), *Parnassia trinervis* NC_043951 (unpublished), *Parnassia palustris* NC_045280 (Yu et al. 2019), *Tribulus terrestris* MN164624 (Yan et al. 2019).

## Ethical approval

Research on plants (either cultivated or wild), including the collection of plant material, was undertaken in compliance with relevant institutional, national, and international guidelines and legislation.

## CRediT authorship statement

Yuan Zhong: Writing – Original Draft, methodology, formal analysis. Jingzheng Zhang: Resources. Zhenzhen Bao: Conceptualization, methodology.

## Data Availability

The genome sequence data that support the findings of this study are openly available in the GenBank of NCBI at [https://www.ncbi.nlm.nih.gov] (https://www.ncbi.nlm.nih.gov/nuccore/OK065822.1/), under the accession no. OK065822. The associated BioProject, SRA, and Bio-Sample numbers are PRJNA761540, SRR15817515, and SAMN21354213, respectively.
